# Unbreakable Resolutions as an Effective Tactic for Self-Control: Lessons From Mahatma Gandhi and a 19th-Century Prussian Prince

**DOI:** 10.3389/fpsyg.2021.771141

**Published:** 2021-12-10

**Authors:** Russell A. Powell, Rodney M. Schmaltz, Jade L. Radke

**Affiliations:** Department of Psychology, MacEwan University, Edmonton, AB, Canada

**Keywords:** unbreakable resolutions, self-control, self-management, Mahatma Gandhi, Prince Pückler-Muskau, delay-discounting, say-do correspondence, willpower

## Abstract

Despite the relative consensus in the self-management literature that personal resolutions are not an effective stand-alone tactic for self-control, some individuals seem capable of using them to exert a remarkable level of control over their behavior. One such individual was Mahatma Gandhi, the famous Indian statesman. Gandhi often used personal resolutions—or “vows”—to commit himself to a range of challenging behaviors, such as extreme diets, sexual abstinence, and fasting. Similarly, Prince Pückler-Muskau, a celebrated 19th-Century adventurer, landscape designer and travel author, described using personal resolutions to unfailingly accomplish numerous tasks in his everyday life. In this article, we examine the historical writings of Gandhi and Pückler-Muskau concerning their use of resolutions. We describe three defining characteristics of their resolutions, which we will refer to as *unbreakable resolutions*, and outline Gandhi’s advice for making and keeping such resolutions. Our analysis suggests that the effectiveness of unbreakable resolutions may be primarily due to the temporally extended contingencies of reinforcement associated with their use, and can be usefully interpreted from the perspective of delay-discounting and say-do correspondence models of self-control. The implications of this examination for understanding the concept of willpower and for enhancing modern research into self-control training are also discussed. Based on this analysis, we additionally offer a tentative set of guidelines on how to make and keep unbreakable resolutions.

## Introduction

As I look back on the 20 years of the vow, I am filled with pleasure and wonderment. The more or less successful practice of self-control (to practice sexual abstinence) had been going on since 1901. But the freedom and joy that came to me after taking the vow had never been experienced before 1906. Before the vow I had been open to being overcome by temptation at any moment. Now the vow was a sure shield against temptation (Gandhi, 1957/1927, p. 208).

Although people frequently use personal resolutions to try to change their behavior, these efforts are often ineffective. For example, while Martatt and Kaplan (1972) found that 75% of college students had managed to keep their New Year’s resolutions after 15 weeks, later studies revealed that only 40% of adults had kept their resolutions after 6 months (Norcross et al., 1989) and only 19% had kept them after 2 years (Norcross and Vangarelli, 1989). Similarly, a study by Wiseman (n.d.) of over 3,000 volunteers found that only 12% had kept their New Year’s resolutions after a year, despite 52% having been confident they would succeed. Consistent with such findings, the self-management literature usually regards a resolution as simply a self-set goal that then requires the use of additional procedures to be achieved (Gollwitzer, 1999; Mischel and Ayduk, 2004; Norcross et al., 2013; Sarafino, 2011; Watson and Tharp, 2014). The many news articles and blog posts that offer advice about New Year’s resolutions [e.g., American Psychological Association, 2019; Miller, 2017] likewise emphasize the need for additional tactics when making a resolution, such as informing others about it, in order to be successful.

In contrast to the general ineffectiveness of resolutions as a stand-alone tactic for self-management, certain individuals seem capable of using resolutions to accomplish very difficult tasks. The famous Indian statesman, Mahatma Gandhi, often used personal resolutions (or “vows”) to commit himself to highly challenging courses of action (Gandhi, 1957/1927, 1930b/2003). These included sexual abstinence, fasting (which he sometimes used as a political tool to inspire his fellow Indians to strive for independence from Britain), and diet (a committed vegetarian, he often experimented with different food restrictions). As will later be discussed, Gandhi also offered advice to others on how to effectively use vows to manage one’s behavior.

Another historical figure who seems to have had an exceptional ability to keep his resolutions, at least by his own account, was Prince Hermann von Pückler-Muskau, a 19th-Century German adventurer, popular travel author, and celebrated landscape designer. As described in James (1907) classic article, *The Energies of Men*:

That delightful being, Prince Pückler-Muskau (1833), writes [to his former wife, Lucie] from England that he has invented a sort of artificial resolution respecting things that are difficult of performance. “My device,” he says, “is this: I give my word of honor most solemnly to myself to do or to leave undone this or that. I am of course extremely cautious in the use of this expedient, but when once the word is given, even though I afterward think I have been precipitate or mistaken, I hold it to be perfectly irrevocable. When the mysterious formula is pronounced, no alteration in my own views, nothing short of physical impossibility, must, for the welfare of my soul, alter my will” (p. 16; see Appendix for the full paragraph from which all Pückler-Muskau quotes in this article are drawn).

As with Gandhi, Pückler-Muskau’s resolutions appear to have functioned as a highly reliable means of controlling his future behavior. (Note that for the purposes of this article, we will adopt the working assumption that both Gandhi’s and Pückler-Muskau’s descriptions of, and opinions about, their resolutions are accurate. This especially pertinent to Pückler-Muskau for whom the paragraph reproduced in the Appendix is our sole source of information concerning his use of resolutions).

When reading about Gandhi’s and Pückler-Muskau’s use of resolutions, the first author of this paper noticed the extent to which these resolutions, which we will refer to as *unbreakable resolutions*, shared certain characteristics that may have contributed to their effectiveness. The present analysis constitutes a close examination of their writings on this issue to determine if they might provide some insight into why such resolutions were effective and how they could be used to reliably manage one’s behavior. Our analysis suggests that the effectiveness of their resolutions may have depended less on the character of the individuals involved—such as the common assumption that Gandhi had tremendous willpower (e.g., Richards, 2005)—and more on the distinctive nature of the resolutions and the contingencies of reinforcement associated with them. The implications of this analysis for how to understand the concept of “willpower” and increase one’s capacity for self-control are also discussed. We also offer a tentative set of guidelines, based on this analysis, on how to make and keep unbreakable resolutions.

## Definitions

For the purposes of this article, a *personal resolution* will be defined as a self-promise (or self-instruction) to control a target behavior that is perceived as otherwise having a low probability of occurring or not occurring as desired. A person who makes a resolution in the morning to go to the gym to exercise later that day believes they are at high risk of not going to the gym and that making the resolution somehow increases the likelihood of going. As so defined, a personal resolution may or may not involve the use of additional self-management tactics to facilitate the desired outcome. For example, one could resolve to go to the gym and, either in addition to the resolution or as an explicit part of the resolution, arrange to meet someone at the gym to increase the likelihood of carrying out the resolution. Personal resolutions can vary greatly in level of difficulty, which would presumably affect not only the difficulty of keeping a resolution but also the types of resolutions one is willing to make.

By comparison, an *unbreakable resolution* can be defined as a type of personal resolution the intention of which (if not always the outcome) is to virtually guarantee the occurrence of the target behavior, the use of additional tactics being perceived as either unnecessary or of secondary importance. Unbreakable resolutions are in this sense a type of *commitment (or precommitment) device*, which can be defined as a self-management tactic that strongly influences the future occurrence of a target behavior, often by severely restricting the availability or attractiveness of alternative behaviors (e.g., Ainslie, 2001; Rachlin, 2000). A common type of commitment device is a behavior contract that enlists the services of another individual to deliver an aversive consequence if the contract is broken (Malott, 1989; Sarafino, 2011). For example, giving a roommate a $20 bill and telling them to keep it if one fails to go to the gym that day would, to the extent that it virtually guarantees that one will go to the gym, be an example of a commitment device. From this perspective, making an unbreakable resolution to go to the gym, if it too guarantees that one will go to the gym, can be viewed as a commitment device that requires only the statement of the resolution to be effective. In similar fashion, in the opening quotation to this article, Gandhi’s vow to practice sexual abstinence seems to have functioned as an unbreakable resolution that effectively committed him to a life of chastity.

## Three Characteristics of Unbreakable Resolutions

Our examination of Gandhi’s and Pückler-Muskau’s writings suggests that the unbreakable resolutions they made shared three major characteristics. First, *their unbreakable resolutions constituted a special class of promises that they perceived as being clearly distinct from other types of promises that exerted weaker control over their behavior.* An example of this distinction can be found in Gandhi’s account of the difficulty he experienced in obtaining his mother’s permission to travel to England to train as a lawyer (Gandhi, 1957/1927). She was concerned that he would violate the family’s religious beliefs by eating meat, drinking liquor, and consorting with women. Gandhi (1957/1927) described the incident as follows:

I said: “Will you not trust me? I shall not lie to you. *I swear* [emphasis added] I shall not touch any of those things…” “I can trust you,” she said, “but how can I trust you in a distant land?” [Eventually, a family advisor] came to my help and said: “I shall get the boy solemnly to take three vows and then he can be allowed to go.” He administered the oath and I vowed not to touch wine, women and meat. This done my mother gave her permission (pp. 38–39).

For both Gandhi and his mother, a “vow” was a much stronger form of commitment than a simple promise, no matter how fervent the promise.

Pückler-Muskau (1833) similarly viewed an unbreakable resolution as highly distinctive, referring to it as his “grand expedient” in which he would give his “word of honor most solemnly” to himself (p. 434). It seems also to have involved some type of special wording, as suggested by his description of it as involving a “*mysterious formula*” (p. 435). As with Gandhi and his vows, Pückler-Muskau seems to have perceived his “grand expedient” as exerting much stronger control over his behavior than other types of promises. The letter to his former wife in which they are described was written toward the end of a 3-year stay in Britain, prior to which he had already admitted to her in other letters that he had broken his promise not to gamble. These failed promises were apparently, in his view, different from the “grand expedient” that he was only now revealing to her.

From a self-management perspective, the distinctive nature of Gandhi’s and Pückler-Muskau’s unbreakable resolutions can be interpreted as serving to create a “bright-line” between self-promises that must be kept and those that allowed for some possibility of not being kept. Creating a “bright-line” between acceptable and unacceptable behaviors, or between circumstances in which a behavior should or should not occur, is a common recommendation in behavior self-management (e.g., Ainslie, 2001; Baumeister and Tierney, 2011). For example, a student who plans to do their math homework between 1:00 and 4:00 in the afternoon has created a much clearer guide for their behavior than a student who vaguely plans to “study sometime today,” with the former having a much higher likelihood of being carried out (Gollwitzer, 1999). Similarly, an unbreakable resolution (or “vow” or “oath”) that *must* be kept constitutes a less ambiguous rule for one’s behavior than a simple promise that one *intends* to keep but recognizes might not be kept. As Gandhi (1929/2003) put it, “a householder, whose watchman says that he would keep watch *as long as he can* (emphasis added), can never sleep in security” (p. 197). The concept of an unbreakable resolution thereby contrasts sharply with the common recommendation that one should expect to, at least occasionally, violate a resolution [e.g., American Psychological Association, 2019; Baumeister and Tierney, 2011]. This recommendation is intended to counter-act what is sometimes called the “what-the-hell effect” (Baumeister and Tierney, 2011)—or in the case of addictive behaviors, the “abstinence (or limit) violation effect” (Marlatt and Gordon, 1985)—in which people tend to completely abandon a resolution or self-change program at the first sign of failure. Unfortunately, while useful in preventing the abandonment of a self-change program, such recommendations also exclude the possibility of a resolution being regarded as unbreakable, thereby foregoing the possible benefits of such resolutions.

*A second characteristic of Gandhi’s and Pückler-Muskau’s unbreakable resolutions is that the act of breaking a resolution was perceived to be highly aversive, independent of the targeted behavior or outcome.* A good example is Gandhi’s (1957/1927) account of a life-threatening illness he once suffered (probably resulting from a dietary experiment in which he had been eating only fruit and nuts). His physician told him that he needed more protein in his diet and urged him to drink milk. Although Gandhi agreed that milk would likely be beneficial, he adamantly refused because it would violate a vow he had once made never to drink milk. Only when confronted with the argument that he probably had in mind the milk of cows and buffalos when he made the vow, and that drinking goat’s milk would therefore not violate the vow, did he relent. But even then, despite recovering his health, he felt exceedingly guilty over sacrificing the spirit, if not the letter, of the vow: “The will to live proved stronger than the devotion to truth. [Nevertheless] the memory of this action even now rankles in my breast and fills me with remorse, and I am constantly thinking how to give up goat’s milk” (p. 455). Pückler-Muskau (1833) likewise seems to have viewed the act of breaking a resolution as extremely aversive: “If I were capable of breaking [a resolution] after such mature consideration, I should lose all respect for myself—and what man of sense would not prefer death to such an alternative?” (p. 435). (Pückler-Muskau also seems to have regarded the act of fulfilling a resolution to be highly reinforcing independent of the targeted outcome: “I… find something very satisfactory in the thought that man has the power of framing such props and such weapons out of the most trivial materials, indeed out of nothing, merely by the force of his will, which hereby truly deserves the name of omnipotent” (p. 435). However, we found no similar statements by Gandhi, possibly because his resolutions were often directed toward long-term goals, the fulfillment of which could be greatly delayed (e.g., India’s independence from Britain, the promotion of non-violence as a means of overcoming oppression, etc.). In other words, many of Gandhi’s resolutions were essentially, throughout his life, “a work in progress”).

The aversive nature of breaking a resolution is largely a function of social conditioning (Kochanska, 1991). For example, Skinner (1953, 1957) defined a resolution as a type of self-instruction that depends on past experiences in which breaking the resolution resulted in aversive consequences from those who knew about it. Through repeated experiences, this “aversive stimulation which leads us to keep the resolution may eventually be supplied automatically by our own behavior [i.e., the covert behavior of “guilty feelings…”], even in the absence of other people” (1953, p. 237). In Gandhi’s case, his mother, whom he described as someone who could “take the hardest vows and keep them without flinching” (Gandhi, 1957/1927, p. 4), would likely have played a major role in this process. As for Pückler-Muskau, his aristocratic upbringing, with its emphasis on the concept of honor—as especially illustrated by his penchant for engaging in sword duels (Bowman, 2010)—may have played a similar role in the development of his aversion to breaking a resolution.

Unfortunately, for most of us, self-induced feelings of guilt are often insufficient to motivate us to do what we resolve to do (Norcross et al., 2013; Steel, 2011), hence, the need for additional incentives to help us keep our resolutions. Skinner (1957), for example, noted that the effectiveness of a resolution “is greater if the resolution is publicly announced or, better, conspicuously posted during the period in which it is in force” (p. 444). Indeed, informing others about one’s resolutions is perhaps the most frequently recommended tactic for enhancing their effectiveness (e.g., American Psychological Association, 2019; Clear, 2018; Levinson and Cooper, 2015). By contrast, Pückler-Muskau seems to have kept his unbreakable resolutions essentially private, first revealing his use of them to his former wife in the letter he wrote to her some 16 years after they were married. And although many of Gandhi’s vows were, often for political reasons, publicly announced, he nevertheless considered a vow to be “a promise made by one to oneself” (Gandhi, 1930a/2003, p. 201). This suggests that there may be additional factors, beyond avoidance of guilt, that underly the effectiveness of unbreakable resolutions.

*A third characteristic of Gandhi’s and Pückler-Muskau’s unbreakable resolutions is that they were regarded as a special device or tool that could be used to obtain a wide variety of future benefits.* Pückler-Muskau (1833), for example, described his resolutions as “a powerful aid in great things as well as in small” (p. 435), which ranged from dealing with interpersonal conflicts —“do you not see that I also possess a formidable weapon of attack, if I were compelled to use it” (p. 433)—to tackling everyday instances of procrastination—“to conquer indolence so as to get vigorously through some long deferred work” (p. 433). Gandhi (1929/2003) similarly regarded the ability to keep vows as having broad application: “The practice of taking vows has come to my rescue in many a crisis; I have seen it save others from many a pitfall” (p. 196). He also believed that “every person (should train themselves) to keep such vows; one can strengthen one’s power of will by doing so and *fit oneself for greater tasks* [emphasis added]” (Gandhi, 1913/2003, p. 192). This view of resolutions as a valuable tool stands in sharp contrast to the modern view of resolutions, at least in Western society, in which they are often regarded as so unreliable as to be considered humorous (e.g., Jeon, 2020) or, by providing false hope, even harmful (e.g., Anagnos, 2019; Murrihy, 2016).

The perception of unbreakable resolutions as a valuable tool may be a key factor underlying their effectiveness. Ainslie (2001), for example, has argued that the efficacy of a self-promise is dependent on the extent to which we have reliably carried out such promises in the past. Breaking a resolution therefore represents not only the loss of the sought-after benefit of that particular resolution, but also the loss of the many other benefits that one might have obtained through the future use of resolutions. This notion will be further elaborated upon in the discussion section.

## Gandhi’s Advice on the Use of Vows

As previously noted, Gandhi sometimes offered advice to others on how to effectively use vows to manage one’s behavior. Much of this advice simply emphasized the need for persistence: for example, “(the person making the vow) should go on striving and never lose heart…. He should banish from his heart the word ‘impossible”’ (Gandhi, 1926a/2003, p. 195). Gandhi also regarded a vow as a spiritual practice; for example, “God is the very image of the vow…. We should, therefore, never doubt the necessity of vows for the purpose of self-purification and self-realization” (Gandhi, 1930b/2003, p. 200). For those who shared such beliefs, the future benefits to be accrued from the use of vows would thereby be perceived as extending into the spiritual realm. Some of his other advice, however, parallels that found in the present-day self-management or self-help literature:

1. *Recognize one’s limitations.* “The taking of vows that are not feasible or that are beyond one’s capacity would betray thoughtlessness and want of balance” (Gandhi, 1929/2003, p. 197). This advice addresses a common problem in behavior self-management, which is that people often try to do too much, thereby placing their self-management attempts at significant risk of failure (Watson and Tharp, 2014).

2. *Start small.* “One may take easy and simple vows to start with and follow them with more difficult ones” (Gandhi, 1913/2003, p. 192). Gandhi especially recommended the use of small vows (called “anuvrat” in Jainism) to abstain from minor bouts of anger and aggression as means of developing the ability to react non-violently in the face of extreme provocation (Kool and Agrawal, 2020a). Gradually increasing the difficulty of a task or target behavior—which is akin to the behavioral concept of shaping and minimizes the likelihood of failure (Martin and Pear, 2011)—is a frequently recommended tactic in behavior self-management (e.g., Fogg, 2020; Guise, 2013; Watson and Tharp, 2014).

*3. Allow for exceptions.* “A vow can be made conditional without losing any of its efficacy or virtue. For instance, there would be nothing wrong about taking a vow to spin for at least one hour every day except when one is travelling or sick” (Gandhi, 1929/2003, p. 197; the spinning of homespun cotton being promoted by Gandhi as a means of undermining the sale of British-made clothing). In other words, one should plan for the possible occurrence of events that could justifiably prevent a vow from being carried out. This includes abandoning a vow if it later becomes apparent that it is in someway wrong or harmful: “If through ignorance one should make any such vow it is one’s duty to break it” (Gandhi, 1926b/2003, p. 193); in fact, such a vow is better described as cancelled rather than broken insofar as “there cannot be a vow to commit a sin” (Gandhi, 1930b/2003, p. 199). Allowing for such exceptions is consistent with Sarafino’s (2011) concept of a “rule-release procedure” that specifies the circumstances under which one can be exempted from the restrictive rules of a self-management contract. The danger, of course, is that allowing for exceptions to vows runs the risk of creating loopholes, a matter that Gandhi was aware of and that required stringent honesty with oneself and clear boundaries between acceptable and unacceptable behaviors to avoid (Kirby, 2013).

4. *If a vow is inadvertently broken, a self-penalty should be imposed to compensate for it*. “If he forgets his vow at any time, he should do *prayaschitta* (penance) and remind himself of the vow” (Gandhi, 1926a/2003, p. 195). To the extent that an act of penance is effortful or unpleasant, it can be conceptualized as an attempt at self-punishment aimed at reducing the likelihood of such violations in the future. However, although self-punishment procedures are sometimes recommended as a self-management tactic (Watson and Tharp, 2014), they are also problematic in that the contingency is entirely under one’s control—hence, nothing prevents one from “short-circuiting the contingency” by carrying out the forbidden behavior and foregoing the punishment (Martin and Pear, 2011; Miltenberger, 2016). With respect to unbreakable resolutions, however, a self-penalty might function as more than a mere attempt at punishment. In keeping with the religious meaning of penance (e.g., Firey, 2008), a promise to penalize oneself for violating a resolution can be viewed as a sort of back-up resolution that enables one to “atone for” or “make reparation for” the failure of the original resolution. To the extent that the reliability of one’s resolutions is perceived to be of critical importance to their effectiveness (Ainslie, 2001), then the act of carrying out the penalty will be positively reinforced by the perceived restoration of that reliability. In the same way that a craftsperson is strongly incentivized to repair a tool that is critical to accomplishing their trade, so too a person who perceives their ability to make and keep resolutions as highly beneficial will be strongly incentivized to make reparation for any violation of a resolution.

5. *For vows that are particularly challenging, additional procedures can be implemented to facilitate keeping them.* Gandhi did not explicitly recommend the use of such procedures as a general strategy; he did, however, contend that fasting and avoidance of certain foods were necessary with respect to practicing sexual abstinence (Gandhi, 1957/1927). It seems likely, therefore, that he would have encouraged the use of additional tactics to assist in keeping other types of difficult resolutions. Although this might seem inconsistent with Gandhi’s contention that a vow must never be broken—which seems to imply that additional self-management tactics should not be needed in order to keep a vow—the use of additional tactics is not inconsistent if, in keeping with the first guideline, they are viewed as increasing one’s capacity to undertake a difficult vow. In other words, what Gandhi may be saying here is that anyone who wishes to take a vow of chastity should do so only if they are also capable of fasting and avoiding certain foods (which, in Gandhi’s case, also involved the use of vows). To take on a vow of chastity in the absence of these practices would, to use his words, “betray thoughtlessness and want of balance” (Gandhi, 1929/2003, p. 197). Along these lines, Gandhi also believed strongly in the importance of adequate sleep (Gandhi, 1905a/2003) and in the practice of effective time-management (Gandhi, 1905b/2003), which would presumably likewise facilitate a person’s ability and willingness to undertake a difficult vow (see also Kapoor, 2017).

The advice Gandhi provided on the use of unbreakable resolutions can be interpreted as serving to reduce or eliminate the risk of a resolution being broken, thereby preserving the integrity of such resolutions as a means of reliably managing one’s behavior. We found no evidence, however, of similar advice in Pückler-Muskau (1833) description of his use of resolutions. He instead seems to have practiced a type of brinksmanship in which the only justifiable excuse for breaking a resolution was “physical impossibility.” His only strategy for mitigating this risk was therefore to be “extremely cautious” and “exercise great deliberation” before making a resolution. Consistent with this strategy, we also found no indication that he used his resolutions to make the type of long-lasting changes in his life that Gandhi did. As noted, for example, he seems not to have used his “grand expedient” to prevent himself from gambling, possibly because he perceived such a resolution as having too high a risk of failure. He may instead have focused on using his resolutions to accomplish less challenging, shorter-term goals including, as previously noted, overcoming everyday instances of procrastination.

## Discussion

The following discussion will present two theoretical models, a delay discounting model and a say-do correspondence model, that help account for the effectiveness of unbreakable resolutions. We will also present some implications of the say-do correspondence model for the popular strength theory of self-control and related modern-day research on self-control training (e.g., Baumeister and Tierney, 2011). This will be followed by the presentation of a brief set of tentative guidelines, based on the present analysis, for using unbreakable resolutions to effectively manage one’s behavior.

### Unbreakable Resolutions: Theoretical Models and Implications

The effectiveness of unbreakable resolutions can partially be accounted for in terms of a delay discounting model of self-control (e.g., Rachlin, 2000; Ainslie, 2001; Kirby, 2013; Ashe and Wilson, 2020; Kool and Agrawal, 2020a). In a simplified version of this model, self-control is defined as the act of choosing a larger later reward (LLR)—a delayed or long-term goal that we highly value—over a smaller sooner reward (SSR)—a relatively immediate, short-term temptation that can potentially interfere with obtaining the LLR. A frequent assumption in delay discounting models is that the value of a reward is a hyperbolic function of its delay, in which reward value increases more and more rapidly as the availability of the reward draws near. It is this hyperbolic increase in reward value that accounts for the common problem of preference reversal, in which we switch preference from the LLR (long-term goal) to the SSR (short-term temptation) when the value of the latter sharply rises as it becomes imminent. From this perspective, the various strategies and tactics of behavior self-management, including the use of resolutions, are intended to prevent preference reversal from occurring.

A deficiency with this simplistic delay discounting model, however, is that the major self-control dilemmas we face typically involve not a singular choice between an SSR and an LLR, but a series of choices that involve an ongoing stream of SSRs, each choice of which is largely inconsequential with respect to the attainment of the LLR (Malott, 1989). For example, would a student really forego studying one evening to play computer games if the student knew that that one evening of gaming would for sure result in a poor grade at the end of the semester? And would anyone indulge in a delicious dessert at the end of a meal if the person knew that that one dessert would for sure result in a heart attack 20 years from now? Rather, it is the inconsequential nature of each choice of an SSR that allows its value, when imminent, to sharply rise above the value of the LLR—often accompanied by the rationalization that one is indulging in the temptation “just this once” (Powell et al., 2017). The problem of course is that one may be repeatedly faced with the same choice, and can make the same rationalization, day after day until the SSR has so often been chosen that the cumulative effect of those choices does undermine one’s ability to obtain the LLR.

An unbreakable resolution, however, forestalls the possibility of this scenario occurring. “Just this once” is not a viable excuse for violating an unbreakable resolution since, by definition, an unbreakable resolution must never be broken (or, in keeping with Gandhi’s advice, never broken without sufficient justification or without being atoned for through penance). Indulging in even a single SSR represents a clear failure of the resolution and the potential loss of not only the sought-after LLR of that particular resolution but also the many other LLRs—“great things as well as small” to use Pückler-Muskau (1833) words—one might have obtained through the future use of such resolutions. Consistent with this interpretation, in what has been termed choice bundling (Ainslie, 2001, 2007; Ainslie et al., 2018), hyperbolic discount functions have been shown to predict increased preference for LLRs over SSRs when one is making a choice, not between a single SSR and a single LLR, but between a series of SSRs and a series of LLRs. For example, Kirby and Guastello (2001) presented students with five weekly choices of a small amount of money that was immediately available vs. a large amount of money that was available 1 week later. The students chose the larger amounts more often if at the start they made a single “bundled” choice for all 5 weeks than if they made separate choices on a weekly basis. Rachlin (2000, 2016) has proposed a similar model in which self-control is viewed as an overall pattern of behavior that is directed toward temporally extended contingencies of reinforcement. From this perspective, the critical incentive that maintains an unbreakable resolution is the temporally extended (or “bundled”) series of long-term benefits that one can potentially obtain through the future use of such resolutions, but only if such resolutions have always been kept (For a detailed description of these more sophisticated delay discounting models directly applied to Gandhi’s use of vows, see Kirby, 2013, and Kool and Agrawal, 2020a).

The present analysis also suggests that the ability to use resolutions in this manner can be conceptualized as a type of “say-do correspondence” (SDC), in which what we say we will do at one point in time matches what we actually do at a later point in time. Training in SDC has been theorized to contribute to the development of self-regulation in children; a child who learns to do what they tell others they will do, such as promising to follow instructions they have been given, will then generalize that ability to doing what they tell themselves they will do, that is, to follow instructions they give to themselves (Anderson and Merrett, 1997; Guevremont et al., 1986; Merrett and Merrett, 1997). Consistent with this SDC model of self-regulation, Howell et al. (2006) found that students’ ratings on a say-do (or self-promises) scale—the three items of which consisted of “to what extent do you keep your promises to yourself even if later on you don’t feel like doing what you had promised yourself to do?” “to what extent do you keep your promises to yourself even when other people are unaware of your promises and only you know about them?” and “to what extent do you use the act of making a promise to yourself to accomplish certain tasks?”—was a stronger predictor of academic procrastination than two standardized measures of procrastination (Soloman and Rothblum, 1984; Tuckman, 1991), a measure of Perceived Academic Control (Perry et al., 2001), and students’ self-reported use of implementation intentions for studying (plans that specify what, where and when they will be studying; Gollwitzer, 1999). A later study by Powell and Howell (2008) additionally found that students’ ratings of their ability to keep self-promises was positively associated with their ratings on measures of self-control, conscientiousness, and flourishing. The results of these studies, though only correlational, are consistent with the possibility that self-promises might, for some individuals, play an important role in their ability to resist temptations and attain long-term goals, with unbreakable resolutions representing an extreme example of this ability.

The present analysis also has implications for understanding the concept of willpower. The popular *strength (or limited-resource) model* of self-control views willpower as a type of internal energy that enables one to resist temptations and effectively regulate one’s behavior (Baumeister et al., 1998; Baumeister and Tierney, 2011). Fluctuations in the available amount of this energy account for the way in which our ability to resist temptations seems to vary over time and across situations. For example, people tend to have more self-control in the morning than in the evening (Kouchaki and Smith, 2014) and less self-control following performance of a difficult task than an easy task (Muraven and Baumeister, 2000; Baumeister et al., 2006). Although aspects of the strength model have been called into question (e.g., Job et al., 2010; Hagger et al., 2016; Friese et al., 2019), Baumeister et al. (2018) maintain that it is still the most comprehensive explanation for many aspects of self-control.

From an SDC perspective, however, willpower can alternately, or additionally, be defined as a type of autonomous self-commitment device that overrides any fluctuations in our ability to resist temptations at later points in time. The difference between an SDC and a strength model conceptualization of willpower can be seen in Baumeister and Tierney’s (2011) interpretation of a resolution made by famed, 19th-Century explorer Henry Morton Stanley. Stanley is best known for his expedition to Africa to find the missionary David Livingstone. The search, however, proved to be extremely difficult, involving severe hardships that few people could have endured. During one particularly difficult stretch, Stanley wrote the following note to himself:

I have taken a solemn oath, an oath to be kept while the least hope of life remains in me, not to be tempted to break the resolution I have formed, never to give up the search, until I find Livingstone alive, or find his dead body (Stanley, 1872, pp. 308–309), as cited in Baumeister and Tierney (2011), p. 150.

Baumeister and Tierney interpreted this oath—which was mirrored by Stanley’s repeated promises in letters and newspaper dispatches to never quit—as a public commitment that served to remove any temptation to abandon the search, the resultant public humiliation being an unacceptable outcome for a person of Stanley’s character and aspirations. By eliminating any temptation to give up, Stanley was thereby able to conserve his “willpower” for overcoming the day-to-day difficulties he would encounter.

The present analysis, however, suggests that Stanley’s oath may have been more than just a public commitment. If Stanley had a long history of reliably carrying out his personal resolutions, and if those resolutions had, in the past, proven highly effective in accomplishing difficult tasks, then the act of making the oath would by itself have served as a strong form of commitment independent of any public awareness of it. In support of this interpretation, Stanley appears to have been a strongly rule-bound individual, a prime example being his habit of carefully shaving each morning, however, grim the situation he might be facing. Baumeister and Tierney (2011) interpreted this habit as an example of Stanley’s use of routine to automatize his behavior, which would again help to conserve willpower that he could apply to other tasks. But it can also be interpreted from an SDC perspective as simply a demonstration of Stanley’s ability to keep his resolutions whatever difficulties he may be facing.

The present analysis also has implications for recent research on self-control training. The strength model of self-control presumes that self-control can, like a muscle, be strengthened through repeated attempts at resisting a temptation or carrying out an effortful activity (e.g., Muraven et al., 1999; Baumeister et al., 2006; Oaten and Cheng, 2006; Muraven, 2010). An SDC model similarly assumes that our ability to keep resolutions can be deliberately trained but does so through strengthening the correspondence between what we tell ourselves we will do and what we actually do. This has relevance for the types of tasks that should be utilized in such training. Recent research on self-control training typically involves participants being assigned some type of effortful task, such as repeatedly using one’s non-dominant hand (e.g., Lee and Kemmelmeier, 2017) or trying to maintain a straight posture throughout the day (e.g., Muraven et al., 1999). In doing so, however, participants are following the instructions of the experimenters—that is, they are doing what *others* have told them to do. From an SDC perspective, self-control training should ideally utilize tasks that are self-selected so that participants are doing what they tell themselves they will do. Interestingly, this was a specific recommendation of Barrett (1915) who, in his classic work on training the will, strongly emphasized the need for trainees to self-select the exercises to be used. Congruent with an SDC model, Barrett also strongly recommended the use of “useless exercises,” such as standing on a chair for 5 min or slowly turning the pages of a 200-page book, thereby maximizing the extent to which the behavior is perceived as being carried out for the sole purpose of following the resolution one had made. Although Barrett’s work on training the will has been essentially ignored by modern researchers—a Google search revealed zero citations of Barrett’s work in the modern self-control literature—variables such as these may be fruitful avenues for further research. This is especially the case given that recent reviews of the research on self-control training have revealed inconsistent evidence for the efficacy of such training (Inzlicht and Berkman, 2015; Beames et al., 2017; Friese et al., 2017).

### Some Tentative Guidelines for the Effective Use of Unbreakable Resolutions

Toward the end of his analysis of the efficacy of Gandhi’s vows from a delay discounting perspective, Kirby (2013) laments that “unfortunately, understanding the theory does not make self-control any easier. Adherence to private vows requires the motivation to resist temptation during our weakest moments” (p. 537). Based on the present analysis, however, we believe that a major difficulty we have in keeping our vows is not so much a matter of fluctuations in motivation than the often inconsequential effects of each single violation (the previously discussed “just this once” rationalization) and the long history that most of us have in breaking our self-promises. As a result, our resolutions are from the outset regarded as unreliable and thereby highly susceptible to being violated when confronted with a significant temptation. If so, the solution to this problem may be to create an entirely new category of resolutions, one that is distinctly different from past resolutions and which are formulated in a way that maximizes the likelihood of the resolution never being broken—or if broken, then done so only under justifiable circumstances or with some type of restitution being made in the form of a self-penalty. To this end, based on the present analysis as well as Gandhi’s advice on the effective use of vows, we have developed a tentative set of guidelines for making unbreakable resolutions. These include: (1) unbreakable resolutions need to be highly distinctive (writing them down in a special notebook—or what some have referred to as a “book of oaths”—has proven effective in this regard, the basic rule being that whatever resolution is written in the notebook *must* be carried out); (2) start with one or two easy, short-term tasks to begin with (e.g., going for a walk in the evening or making a phone call that one has been postponing) and gradually increase their level of difficulty; (3) incorporate a rule-release procedure that allows for circumstances in which suspending or abandoning a resolution would be justified (with careful consideration given to the danger of creating a loophole); (4) impose a penalty on oneself if a resolution has been broken without sufficient justification, even if that determination is made at a much later point in time (taking a cold shower, foregoing coffee for a day, and throwing away money are examples that have been used); and (5) consider the use of additional self-management tactics when making a resolution to accomplish a particularly difficult task. Two additional recommendations we have made are to, at least initially, not tell others about one’s resolutions—the goal being to demonstrate to oneself the ability to keep a resolution in the absence of any social pressure to do so (this too being a recommendation by Barrett, 1915)—and to treat the use of resolutions as an exercise in self-experimentation, since the precise ways in which these guidelines are best applied are likely to vary across individuals. Preliminary evidence suggests that these guidelines may be worthy of further investigation, with at least some individuals finding them to be surprisingly effective (Powell et al., 2020).

Finally, although unbreakable resolutions might at first glance seem to be the ultimate self-control device—requiring only a verbal promise to oneself—they should not be regarded as a panacea for managing one’s behavior. For one thing, similar to other types of commitment devices such as behavioral contracts, there may be considerable variability across individuals, as well as over time and across situations, in the extent to which one is willing to make an unbreakable resolution. Despite the extent to which a resolution may be effective in overriding short-term fluctuations in motivation, very difficult resolutions will likely require very high and consistent levels of motivation before they will even be attempted. Pückler-Muskau is perhaps a good example in this regard. The trip to England during which he wrote the letter describing his use of resolutions was made for the express purpose of finding a wealthy British heiress to marry (a plan that his wife, Lucie, helped devise and the reason for which they got a divorce). Through careless spending, including an addiction to gambling, he had squandered most of his inheritance, apparently having made little or no attempt to use his “grand expedient” to get his spending under control. As earlier mentioned, this may be because he recognized that there was too great a risk of the resolution being broken, but it might also be because he enjoyed gambling too much to fully commit himself to not gamble. As for Gandhi, his reputation has been tarnished by the recent revelation of his practice of sleeping naked with young women as a “yogic” test of his ability to resist sexual temptations (Lal, 2000)—which sounds dangerously close to being a convenient rationalization for indulging in sexual behavior and of which those close to him were highly critical of and attempted to hide from the public. There is therefore no guarantee that unbreakable resolutions will be effectively used or used for appropriate purposes.

Unbreakable resolutions are therefore perhaps best regarded as simply another self-management tactic, one that has been largely overlooked in the present-day literature on self-control and self-management, that some people may wish to consider including in their armamentarium of tactics. That said, however, such resolutions might also, for at least some individuals, constitute a particularly effective means of accomplishing very difficult goals. Consistent with this possibility, Kool and Agrawal (2020a,b) have argued that Gandhi’s success in creating major changes at a societal level, in which the effective use of vows was considered an important tool, may provide important lessons for how the world can best meet the many challenges, including ecological challenges, it is now facing. If so, the present article will hopefully contribute to that endeavor.

## Data Availability Statement

The original contributions presented in the study are included in the article/supplementary material, further inquiries can be directed to the corresponding author/s.

## Author Contributions

RP conceived of the main concept under discussion and the underlying theoretical basis for its effectiveness. RS and JR helped to elaborate upon the concept and its theoretical basis, participated in the development of the suggested guidelines, and contributed to writing the article. All authors have read the manuscript in its submitted form and agree to be accountable for the content.

## Conflict of Interest

The authors declare that the research was conducted in the absence of any commercial or financial relationships that could be construed as a potential conflict of interest.

## Publisher’s Note

All claims expressed in this article are solely those of the authors and do not necessarily represent those of their affiliated organizations, or those of the publisher, the editors and the reviewers. Any product that may be evaluated in this article, or claim that may be made by its manufacturer, is not guaranteed or endorsed by the publisher.

## Appendix

[From Letter XLI, written by Prince Hermann Pückler-Muskau on November 19, 1828, to his former wife, Dowager Countess Lucie von Pappenheim]

To-day I found myself compelled to do something which was very disagreeable to me, and which I had long deferred; I was obliged to resort to my “grand expedient,” in order to conquer my aversion. You will laugh when I tell you what this is; but I find it a powerful aid in great things as well as in small. The truth is, there are few men who are not sometimes capricious, and yet oftener vacillating. Finding that I am not better than others in this respect, I invented a remedy of my own, a sort of artificial resolution respecting things which are difficult of performance,—a means of securing that firmness in myself which I might otherwise want, and which man is generally obliged to sustain by some external prop. My device then is this:—I give my word of honor most solemnly to myself, to do, or to leave undone, this or that. I am of course extremely cautious and discreet in the use of this expedient, and exercise great deliberation before I resolve upon it; but when once it is done, even if I afterward think I have been precipitate or mistaken, I hold it to be perfectly irrevocable, whatever inconveniences I foresee likely to result. And I feel great satisfaction and tranquility in being subject to such an immutable law. If I were capable of breaking it after such mature consideration, I should lose all respect for myself;—and what man of sense would not prefer death to such an alternative? for death is only a necessity of nature, and consequently not an evil; it appears to us so only in connection with our present existence; that is to say, the instinct of self-preservation recoils from death; but reason, which is eternal, sees it in its true form, as a mere transition from one state to another. But a conviction of one’s own unconquerable weakness is a feeling which must embitter the whole of life. It is therefore better, if it comes to the struggle, to give up existence for the present with a feeling of inward triumph, than to crawl on with a chronic disease of the soul. I am not made dependent by my promise; on the contrary, it is just that which maintains my independence. So long as my persuasion is not firm and complete, the mysterious formula is not pronounced; but when once that has taken place, no alteration in my own views—nothing short of physical impossibility—must, for the welfare of my soul, alter my will. But whilst I thus form to myself a firm support in the most extreme cases, do you not see that I also possess a formidable weapon of attack, if I were compelled to use it, however, small and inconsiderable the means may appear to many? I, on the contrary, find something very satisfactory in the thought, that man has the power of framing such props and such weapons out of the most trivial materials, indeed out of nothing, merely by the force of his will, which hereby truly deserves the name of omnipotent. I cannot answer for it that this reasoning will not appear to you, dear Julia, distorted and blameworthy: indeed it is not made for a woman; while on the other hand a completely powerful mind would perhaps as little stand in need of it. Every man must, however, manage himself according to his own nature; and as no one has yet found the art of making a reed grow like an oak, or a cabbage like a pine-apple, so must men, as the common but wise proverb has it, cut their coat according to their cloth. Happy is he who does not trust himself beyond his strength! But without being so tragical about the matter, this grand expedient is of admirable use in trifles. For example, to fulfill tedious, irksome duties of society with the resignation of a calm victim,—to conquer indolence so as to get vigorously through some long deferred work,—to impose upon oneself some wholesome restraint, and thus heighten one’s enjoyment afterward,—and many, many more such cases, which this occasionally sublime, but generally childish life presents. (Pückler-Muskau, 1833, pp. 434–435).
